# Reassessing the link between adiposity and head and neck cancer: a Mendelian randomization study

**DOI:** 10.1101/2024.11.21.24317707

**Published:** 2025-01-30

**Authors:** Fernanda Morales-Berstein, Jasmine Khouja, Mark Gormley, Elmira Ebrahimi, Shama Virani, James McKay, Paul Brennan, Tom G Richardson, Caroline L Relton, George Davey Smith, M Carolina Borges, Tom Dudding, Rebecca C Richmond

**Affiliations:** 1 MRC Integrative Epidemiology Unit, University of Bristol, Bristol, United Kingdom.; 2 Population Health Sciences, Bristol Medical School, University of Bristol, Bristol, United Kingdom.; 3 University of Bristol Dental School, 1 Trinity Walk, Avon Street, Bristol, United Kingdom.; 4 Genomic Epidemiology Branch, International Agency for Research on Cancer, World Health Organization, Lyon, France.; 5 London School of Hygiene & Tropical Medicine, Keppel Street, London WC1E 7HT, United Kingdom.

**Keywords:** Head and neck cancer, adiposity, body mass index, waist-to-hip ratio, waist circumference, Mendelian randomization

## Abstract

**Background:**

Adiposity has been associated with an increased risk of head and neck cancer (HNC). Although body mass index (BMI) has been inversely associated with HNC risk among smokers, this is likely due to confounding. Previous Mendelian randomization (MR) studies could not fully discount causality between adiposity and HNC due to limited statistical power. Hence, we aimed to revisit this using the largest genome-wide association study (GWAS) of HNC available, which has more granular data on HNC subsites.

**Methods:**

We assessed the genetically predicted effects of BMI (N=806,834), waist-to-hip ratio (WHR; N=697,734) and waist circumference (N=462,166) on the risk of HNC (N=12,264 cases and 19,259 controls) and its subsites (oral, laryngeal, hypopharyngeal and oropharyngeal cancers) using a two-sample MR framework. We used the inverse variance weighted (IVW) MR approach and multiple sensitivity analyses including the weighted median, weighted mode, MR-Egger, MR-PRESSO, and CAUSE approaches. We also used multivariable MR (MVMR) to explore the direct effects of the adiposity measures on HNC, while accounting for smoking behaviour, a well-known HNC risk factor.

**Results:**

In univariable MR, higher genetically predicted BMI increased the risk of overall HNC (IVW OR=1.17 per 1 standard deviation [1-SD] higher BMI, 95% CI 1.02–1.34, p=0.03), with no heterogeneity across subsites (Q p=0.78). However, the effect was not consistent in sensitivity analyses. The IVW effect was attenuated when smoking was included in the MVMR model (OR accounting for comprehensive smoking index=0.96 per 1-SD higher BMI, 95% CI 0.80–1.15, p=0.64) and CAUSE indicated the IVW results could be biased by correlated pleiotropy. Furthermore, we did not find a link between genetically predicted WHR (IVW OR=1.05 per 1-SD higher WHR, 95% CI 0.89–1.24, p=0.53) or waist circumference and HNC risk (IVW OR=1.01 per 1-SD higher waist circumference, 95% CI 0.85–1.21, p=0.87).

**Conclusions:**

Our findings suggest that adiposity does not play a role in HNC risk.

## Introduction

Head and neck cancer (HNC) is among the ten most common cancers in Europe, with an age standardised incidence rate of 10.3 per 100,000 person-years[1]. Around 90% of HNCs are classed as squamous cell carcinomas of the oral cavity, pharynx or larynx[2]. Tobacco smoking and alcohol consumption are well-established HNC risk factors[3–9]. High-risk human papillomavirus (HPV) infection has also been causally linked to the risk of HNC, especially oropharyngeal cancer[10–12]. In contrast, the role of adiposity in the development of HNC is less well understood.

The World Cancer Research Fund/American Institute for Cancer Research (WCRF/AICR) Continuous Update Project (CUP) Expert Report published in 2018 determined higher body fatness (i.e., body mass index [BMI], waist-to-hip ratio [WHR] and waist circumference) likely increases the risk of HNC[13]. The CUP panel reached this conclusion even though a higher BMI has been associated with a decreased risk of HNC[13], since they noted the inverse association appears to be limited to current smokers. They concluded the association between BMI and HNC risk may be biased among individuals who smoke (because smoking is a HNC risk factor associated with lower weight). It is thought that nicotine consumption could lead to appetite suppression and increased energy expenditure, which could in turn, lead to weight loss (and spurious inverse associations between BMI and HNC risk[14]) among smokers[15]. Among never smokers, BMI has been positively associated with HNC risk, in line with the evidence observed for measures of central adiposity (i.e., WHR and waist circumference)[14, 16].

However, the relationship between adiposity and smoking is complex, with evidence from Mendelian randomization (MR) studies suggesting higher adiposity increases the risk of smoking[17, 18] while simultaneously suggesting smoking may lead to lower adiposity[17, 19–22]. Additionally, excess adiposity, socioeconomic deprivation and (both active and passive) smoking are often strongly correlated[23–26]. Thus, the positive associations between adiposity (i.e., BMI among non-smokers, WHR and waist circumference) and HNC risk may not be as unbiased as they appear.

It is important to acknowledge that previous MR studies on adiposity (i.e., BMI, WHR, waist circumference) and HNC risk were relatively small (maximum N=6,034 cases) and could not fully discount causality due to limited statistical power[27–29]. Therefore, the aim of this MR study was to revisit the link between adiposity and HNC risk using data from a HNC genome-wide association study (GWAS) that includes over two times the number of cases than the Genetic Associations and Mechanisms in Oncology (GAME-ON) GWAS[30] used by Gormley et al.[28] (the largest to date; N=12,619, including 6,034 cases and 6,585 controls) and has more granular data on HNC subsites (i.e., oral cavity, hypopharynx, oropharynx and larynx). We also aimed to use multivariable MR (MVMR) to explore the direct effects of the adiposity measures on HNC, while accounting for smoking behaviour.

## Methods

### Study design

We used a two-sample MR framework to assess the genetically predicted effects of BMI, WHR and waist circumference on the risk of HNC and its subsites (oral, laryngeal, hypopharyngeal and oropharyngeal cancers) among individuals of European ancestry. Genetic variants associated with these adiposity traits were used as instrumental variables to estimate causal effects under the three core MR assumptions[31]: 1) the genetic variants are strongly associated with the adiposity trait of interest (relevance assumption); 2) the distribution of the genetic variants in the population is not influenced by factors that also influence HNC risk, such as population stratification, assortative mating and dynastic effects (independence assumption); and 3) the genetic variants can only influence HNC risk via their effect on the adiposity trait of interest (exclusion restriction assumption). This work was conducted and reported according to the STROBE-MR guidelines[32] (Additional File 1: Appendix).

### Head and neck cancer GWAS

GWAS summary statistics for HNC were obtained from a European HEADSpAcE consortium GWAS that excluded UK Biobank participants (N=31,523, including 12,264 cases and 19,259 controls) to avoid overlapping samples across the exposure and outcome datasets. It includes the European GAME-ON data used by Gormley et al.[30] and has more granular data on HNC subsites (i.e., oral cavity [N=21,269, including 3,091 cases and 18,178 controls], hypopharynx [N=18,652, including 474 cases and 18,178 controls], HPV positive oropharynx [N=20,146, including 1,980 cases and 18,166 controls], HPV negative oropharynx [N=19,114, including 948 cases and 18,166 controls] and larynx [N=20,668, including 2,490 cases and 18,178 controls])[33].

HNC was defined based on the 10^th^ revision of the International Classification of Diseases (ICD-10)[34]. It included cancers of the oral cavity (C00.3, C00.4, C00.5, C00.6, C00.8, C00.9, C02.0–C02.9 [except C02.4], C03.0–C03.9, C04.0–C04.9, C05.0–C06.9 [except C05.1, C05.2]), the oropharynx (C01-C01.9, C02.4, C05.1, C05.2, and C09.0–C10.9), the hypopharynx (C12.0-C13.0), the larynx (C32), and overlapping or not otherwise specified sites (C14, C05.8, C02.8, C76.0)[33].

Further detail on the HEADSpAcE GWAS has been published elsewhere[33]. In brief, genotype data were obtained using nine different genotyping arrays. They were subsequently converted to genome build 38 for consistency across datasets. Quality control (QC) procedures were conducted by genotyping array rather than by study. Samples were excluded for the following reasons: sex mismatch (heterozygosity <0.8 for males and >0.2 for females), autosomal heterozygosity (>3 standard deviation [SD] units from the mean), missingness (>0.03), and cryptic relatedness (identity-by-decent >0.185). Single nucleotide polymorphisms (SNPs) were removed due to genotype missingness (>0.01), deviations from Hardy-Weinberg equilibrium (*p* <1e-05) and low minor allele count (<20). Imputation was performed using the TOPMed Imputation Server. Only SNPs with an imputation score r^2^ >0.3 and a minor allele frequency (MAF) >0.005 were included in the GWAS. The analyses were conducted in PLINK using logistic regressions adjusted for sex, the top principal components and imputation batch (six in total, which account for both genotyping array and study differences).

### Genetic instruments for adiposity

GWAS summary statistics for waist circumference (N=462,166) in SD units were obtained from the UK Biobank available via the IEU OpenGWAS platform (id: ukb-b-9405). GWAS summary statistics for BMI (N=806,834) and WHR (N=697,734) in SD units were obtained from the latest Genetic Investigation of Anthropometric Traits (GIANT) consortium’s GWAS meta-analysis by Pulit et al.[35] available at https://zenodo.org/records/1251813. The meta-analysis is the biggest to date, as it combines the meta-analysis by Shungin et al.[36] with UK Biobank data. The UK Biobank GWAS[35] was conducted using imputed data and the BOLT-LMM software[37]. The linear mixed models (LMMs) were solely adjusted for genotyping array. GIANT and UK Biobank data were meta-analysed[35] using an inverse-weighted fixed-effect meta-analysis in METAL[38].

We extracted GWAS-significant SNPs for waist circumference using the standard threshold (p<5e-08). For BMI and WHR, we extracted them according to the stringent threshold recommended by Pulit et al.[35] to account for denser imputation data (p<5e-09). We then performed LD-clumping to select independent lead SNPs for each exposure (r^2^=0.001, 10,000 kb). In total, 458 and 283 and 375 SNPs remained for BMI, WHR and waist circumference, respectively.

### Data harmonisation

We extracted HNC GWAS summary statistics that corresponded to the list of SNPs selected as instruments for the exposures. Proxy SNPs (r^2^>0.8) were used when the instrumental SNPs were not available in the outcome datasets. Proxies were identified using the “extract_outcome_data” function of the “TwoSampleMR” R package and the 1000 Genomes Project European reference panel. We harmonised the exposure and outcome datasets using the “harmonise_data” function of the “TwoSampleMR” R package[39]. Positive strands were inferred using allele frequencies and ambiguous palindromic SNPs with MAFs ≥0.3 were removed. The harmonised data used in the analyses are available in Supplementary Tables 1 to 6 (Additional File 2).

We calculated mean F-statistics and total R^2^ values to assess the strength of our genetic instruments after data harmonisation[40, 41].

### Statistical analysis

#### Main analyses

The multiplicative random effects inverse-variance weighted (IVW) MR approach[42] (the default IVW method of the “TwoSampleMR” package[39]) was used to investigate the genetically predicted effects of BMI, WHR and waist circumference on HNC risk. We did not correct our results for multiple testing, as all our exposures are strongly correlated[35, 36].

#### Sensitivity analyses

Because the IVW method assumes all genetic variants are valid instruments[42], which is unlikely the case, three pleiotropy-robust two-sample MR methods (i.e., MR-Egger[43], weighted median[44] and weighted mode[45]) were used in sensitivity analyses. We also performed tests for SNP heterogeneity (i.e., Q statistic test)[46] and directional horizontal pleiotropy (i.e., MR-Egger intercept test)[43]. When directional horizontal pleiotropy was identified, we used the intercept value to evaluate the extent of the bias. The MR-PRESSO[47] method was used to identify outliers (outlier test p<0.05) and calculate outlier-corrected causal estimates when there was evidence of SNP heterogeneity. The MR-PRESSO distortion test was used to evaluate differences between the outlier-corrected and IVW estimates.

In addition, we ran MVMR[48] analyses to evaluate the direct effects of adiposity measures with evidence of a total effect in our main analyses. The aim of the MVMR analyses was to separate the effect of adiposity from smoking behaviour (a well-known HNC risk factor which has a complex relationship with adiposity) in the development of HNC. We obtained genetic instruments for smoking behaviour from two different sources: a smoking initiation GWAS (N=805,431 excluding 23andme) derived by the GWAS and Sequencing Consortium of Alcohol and Nicotine use (GSCAN)[49] and a comprehensive smoking index (CSI; a measure of lifetime smoking that captures smoking heaviness, duration and cessation) GWAS (N=462,690) conducted by Wootton et al.[50]. Each was separately investigated in a MVMR framework. For each smoking trait, we selected SNPs that passed the GWAS-significance and independence thresholds (p<5e-08, r^2^=0.001, 10,000 kb) and combined them with the list of SNPs identified as instruments for the relevant exposure. We then performed LD-clumping across the combined list of SNPs, to then use these independent SNPs in MVMR analyses. The exposure and outcome datasets were harmonised to the same effect allele using the “harmonise_data” function of the “TwoSampleMR” R package[39]. We formatted the data using the “format_mvmr” function of the “MVMR” R package, calculated the conditional F-statistics for the MVMR instruments using the “strength_mvmr” function and ran the MVMR analyses using the “ivw_mvmr” function.

Since we used large GWAS datasets for the selection of our genetic instruments, our analyses are at an increased likelihood of being biased due to correlated horizontal pleiotropy (sometimes referred to as heritable confounding), which occurs when the genetic instruments are associated with the exposure through their effect on confounders of an exposure-outcome association[51, 52]. To mitigate this bias, we used Steiger filtering[53] to remove SNPs that are more strongly associated with smoking behaviour (a confounder of the exposure-outcome association) than the exposure of interest, as proposed by Sanderson et al.[54]. We also used Causal Analysis using Summary Effect Estimates (CAUSE)[52], another pleiotropy-robust MR method, to further investigate whether our results could be biased by correlated horizontal pleiotropy. CAUSE uses Bayesian expected log pointwise posterior predictive densities (ELPDs) to compare null, sharing and causal models. A higher ELPD represents a better model fit, so a positive delta ELPD (where delta ELPD = ELPD model 1 − ELPD model 2) suggests model 1 fits the data better than model 2, while a negative delta ELPD suggests the opposite. If we find evidence to reject the null hypothesis that the sharing model (i.e. causal effect fixed at zero) fits the data at least as well as the causal model (i.e. causal effect can differ from zero), our findings would be consistent with a causal effect. Steiger filtering and CAUSE analyses were only conducted for adiposity measures with evidence of a total effect in our main IVW analyses.

Moreover, we used the MR-Clust algorithm[55] to find distinct SNP clusters underlying the relationship between adiposity measures with evidence of a total effect in our main analyses and HNC. The identification of substantial clusters could provide insight into potential causal mechanisms. It could also flag pleiotropic variables that are associated with SNPs in each cluster. We filtered SNPs with conditional probabilities <0.8. At least four SNPs needed to remain per cluster for a substantial cluster to be reported.

#### Secondary analyses

In secondary analyses, we investigated the role of BMI, WHR and waist circumference on the risk of HNC by subsite (i.e., oral cavity, hypopharynx, HPV positive oropharynx, HPV negative oropharynx and larynx). We used a Cochran’s Q test to examine heterogeneity across HNC subsites.

We also explored the role of other adiposity-related anthropometric measures on the risk of HNC and its subsites. These anthropometric measures included: 1) four body shape principal components[56], 2) childhood and adulthood body size[57], 3) metabolically favourable and unfavourable adiposity[58], 4) body fat percentage, and 5) brain and adipose tissue-specific BMI[59]. The data sources for these traits are summarised in Table 1.

#### Statistical software

We completed all MR analyses using R software version 4.4.0 and the “TwoSampleMR” v0.6.3, “MRPRESSO” v1.0, “MVMR” v0.4, “cause” v1.2.0 and “mrclust” v0.1.0 R packages. The “ggplot2” v3.5.1 and “ggforestplot” v0.1.0 R packages were used to create forest plots. The code used to run the MR analyses is available at http://github.com/fernandam93/adiposity_HNC_MR.

## Results

### Genetic instruments for BMI, WHR and waist circumference

After data harmonisation and the removal of ambiguous palindromic SNPs, 442 genetic variants remained as instruments for BMI, while 267 remained for WHR and 353 for waist circumference (Additional File 2: Supplementary Table 1). The mean F-statistic for BMI was 77 (range 33–844) and the total variance explained was 4.8%. For WHR, the mean F-statistic was 73 (range 33–820) and the total variance explained was 3.1%. For waist circumference, the mean F-statistic was 58 (range 30–940) and the total variance explained was 4.4%.

The F-statistics and R^2^ values for the other adiposity-related anthropometric measures have been summarised in Table 2.

### Genetically predicted effects of BMI, WHR and waist circumference on HNC risk

**Figure 1.**
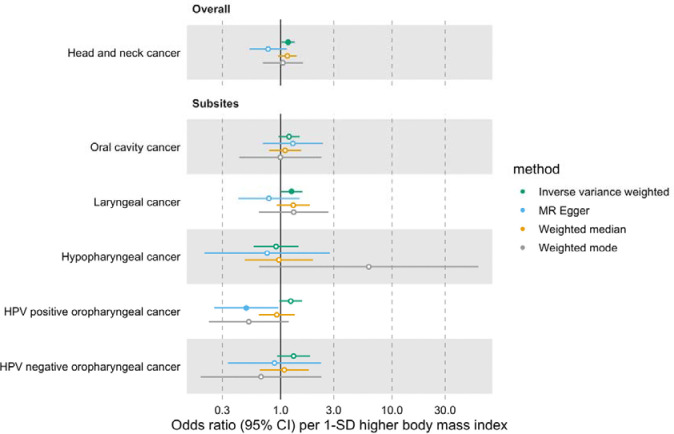
Forest plot for the genetically predicted effects of body mass index on the risk of head and neck cancer and its subsites.

In univariable MR, higher genetically predicted BMI increased the risk of overall HNC (IVW OR=1.17 per 1 standard deviation [1-SD] higher BMI, 95% CI 1.02–1.34, p=0.03), with no heterogeneity across subsites (Q p=0.78) (Figure 1, Supplementary Figure 1 and Additional File 2: Supplementary Table 7). However, the positive relationship between genetically predicted BMI and HNC risk was not consistent across the MR-Egger, weighted median and weighted mode analyses, with point estimates in opposing directions and confidence intervals including the null. The Q statistic and MR-Egger intercept tests suggested that there was heterogeneity across individual SNP estimates (Q=609, p<0.001) and a minor degree of unbalanced horizontal pleiotropy (intercept=0.007, p=0.03) that could have biased the main IVW results (Additional File 2: Supplementary Tables 8 and 9). Although the MR-PRESSO analysis identified two outliers (i.e., rs11611246 and rs9603697), the distortion test suggested the outlier-corrected estimate (outlier-corrected IVW OR=1.14 per 1-SD higher BMI, 95% CI 1.00–1.30, p=0.05) was not statistically different to the main IVW estimate (p=0.94) (Additional File 2: Supplementary Table 10).

**Figure 2.**
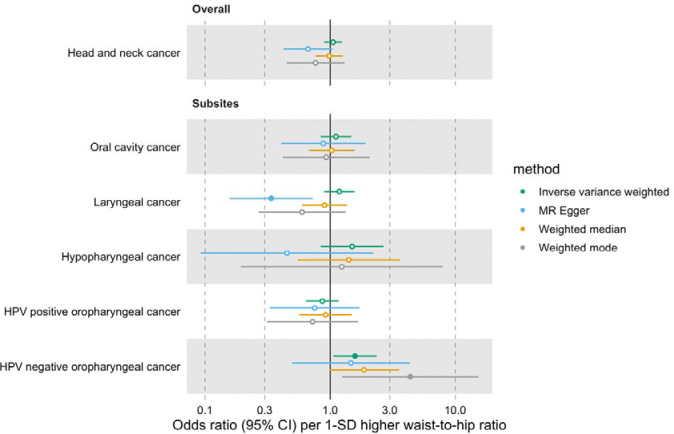
Forest plot for the genetically predicted effects of waist-to-hip ratio on the risk of head and neck cancer and its subsites.

Furthermore, we did not find a link between genetically predicted WHR and HNC risk (IVW OR=1.05 per 1-SD higher WHR, 95% CI 0.89–1.24, p=0.53) and there was no heterogeneity across subsites (Q p=0.15) (Figure 2, Supplementary Figure 2 and Additional File 2: Supplementary Table 7). MR-Egger, weighted median and weighted mode results were consistent with a null effect. The Q statistic and MR-Egger intercept tests suggested that there was some evidence of SNP heterogeneity (Q=332, p=0.004) and unbalanced horizontal pleiotropy (intercept=0.008, p=0.03) (Additional File 2: Supplementary Tables 8 and 9). The MR-PRESSO analysis did not identify any significant outliers (Additional File 2: Supplementary Table 10).

**Figure 3.**
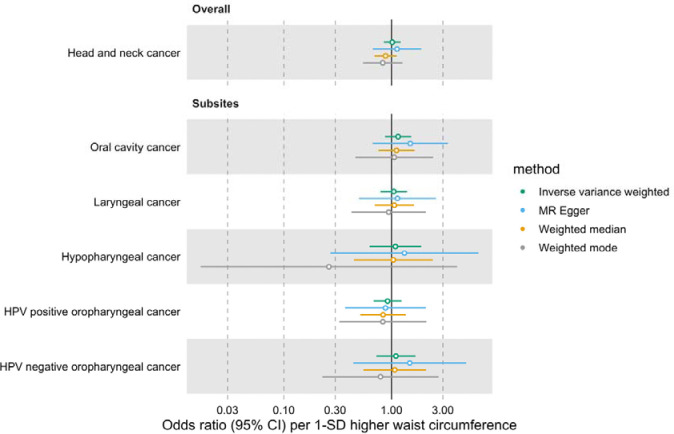
Forest plot for the genetically predicted effects of waist circumference on the risk of head and neck cancer and its subsites.

Similarly, we did not find a genetically predicted effect of waist circumference on HNC risk (IVW OR=1.01 per 1-SD higher waist circumference, 95% CI 0.85–1.21, p=0.87) or evidence of heterogeneity across subsites (Q p=0.87) (Figure 3, Supplementary Figure 3 and Additional File 2: Supplementary Table 7). The MR-Egger, weighted median and weighted mode consistently suggested the absence of an effect of waist circumference on HNC risk. The Q statistic and MR-Egger intercept tests suggested SNP heterogeneity (Q=563, p<0.001) but no unbalanced horizontal pleiotropy (intercept=−0.002, p=0.68) (Additional File 2: Supplementary Tables 8 and 9). The MR-PRESSO analysis identified four outliers (i.e., rs1229984, rs1336486, rs17446091, and rs55726687) but the distortion test suggested the outlier-corrected estimate (outlier-corrected IVW OR=0.98 per 1-SD higher waist circumference, 95% CI 0.84–1.15, p=0.82) was not statistically different to the main IVW estimate (p=0.12) (Additional File 2: Supplementary Table 10).

### MVMR estimates for BMI on HNC risk after accounting for smoking behaviour

**Figure 4.**
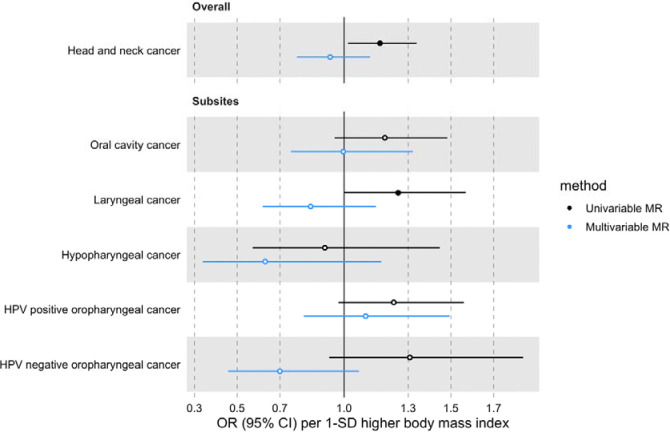
Forest plot for the genetically predicted effects of BMI on the risk of HNC and its subsites, before (univariable-black) and after (multivariable-blue) accounting for comprehensive smoking index (CSI).

**Figure 5.**
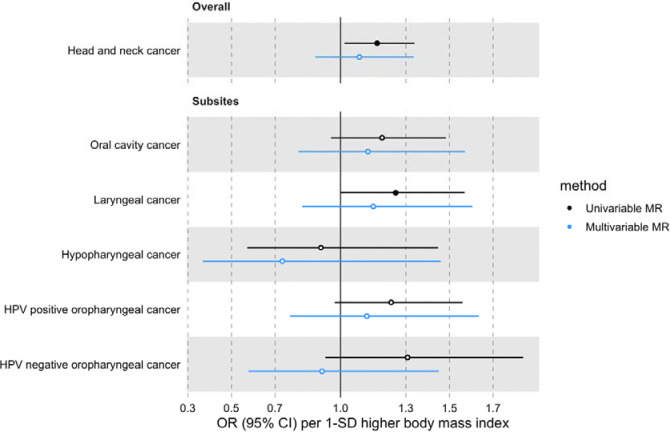
Forest plot for the genetically predicted effects of BMI on the risk of HNC and its subsites, before (univariable-black) and after (multivariable-blue) accounting for smoking initiation (SI).

The effect of BMI on HNC risk was attenuated when smoking behaviour was included in the MVMR model (OR accounting for CSI=0.93 per 1-SD higher BMI, 95% CI 0.78–1.12, p=0.47; OR accounting for smoking initiation=1.09 per 1-SD higher BMI, 95% CI 0.88–1.34, p=0.43) (Figures 4 and 5 and Additional File 2: Supplementary Table 11). Genetically predicted smoking behaviour increased the risk of HNC even after accounting for BMI (CSI OR accounting for BMI=4.25 per 1-SD higher CSI, 95% CI 3.18–5.67, p<0.001; SI OR accounting for BMI=2.10 per 1-SD higher SI, 95% CI 1.61–2.73, p<0.001). The conditional F-statistics for the BMI estimates were 30.5 and 30.3 in the CSI and smoking initiation analyses, respectively (Additional File 2: Supplementary Table 11). They were slightly lower for the smoking behaviour estimates conditioning on BMI (13.4 and 19.5 in the CSI and smoking initiation analyses, respectively).

### MR estimate for BMI on HNC risk after Steiger filtering SNPs more strongly associated with smoking behaviour than BMI

After removing six SNPs (i.e., rs10002111, rs2503185, rs264941, rs10858334, rs225882, rs2273175) that were more strongly associated with smoking behaviour (i.e., CSI or smoking initiation) than BMI, the genetically predicted effect of BMI on HNC risk slightly attenuated towards the null (Steiger filtered IVW OR=1.14 per 1-SD higher BMI, 95% CI 1.00–1.31, p=0.05) (Additional File 2: Supplementary Table 12).

### CAUSE estimate for BMI on HNC risk

We did not find evidence against bias due to correlated pleiotropy, since the causal model did not fit the data much better than the sharing model (CAUSE OR 1.12 per 1-SD higher BMI, 95% credible interval 0.93–1.34, delta ELPD for sharing vs causal=−0.07, *p*=0.47) (Supplementary Figure 4). Interestingly, neither the sharing nor the causal model fitted the data much better than the null model (delta ELPD for null vs sharing=−0.39, *p*=0.36; and delta ELPD for null vs causal=−0.46, *p*=0.41).

### MR-Clust estimates for the relationship between BMI and HNC risk

After filtering SNPs with conditional probabilities <0.8 and clusters with fewer than four SNPs (e.g., cluster 1, as only three of 17 SNPs remained after probability filtering), only a null cluster including 372 SNPs (424 before filtering) remained in the MR-Clust output for BMI and HNC risk (Supplementary Figure 5 and Additional File 2: Supplementary Table 13). Hence, the MR-Clust analysis did not reveal any mechanistic pathways underlying the effect observed.

### Genetically predicted effects of other adiposity-related anthropometric measures on HNC risk

We did not find consistent evidence of genetically predicted effects of other anthropometric measures on HNC risk (Supplementary Figures 6–10 and Additional File 2: Supplementary Table 14). The IVW estimate for PC2 capturing a combination of taller height and slimmer waist suggested this body shape decreased HNC risk (OR=0.86, 95% CI 0.75–0.99, p=0.04) (Supplementary Figure 6b). Similarly, the IVW estimate for PC3 capturing a combination of taller height and narrower hips suggested this body shape also reduced HNC risk (OR=0.73, 95% CI 0.55–0.97, p=0.03) (Supplementary Figure 6c). However, these inverse relationships were not consistent with results obtained using pleiotropy-robust methods (i.e., MR-Egger, weighted median and weighted mode).

## Discussion

In this MR study, we reaffirmed that there is no clear evidence of a genetically predicted effect of adiposity (i.e., BMI, WHR and waist circumference) or related anthropometric measures on the risk of HNC or its subsites. Although we found higher genetically predicted BMI increased the risk of overall HNC in the main univariable MR analysis, this was not consistent across the sensitivity analyses. Notably, the MVMR results suggested the main analysis may have been biased by smoking (and/or related traits), as the effect disappeared after accounting for smoking behaviour. The results obtained after Steiger filtering SNPs more strongly associated with smoking behaviour than BMI suggested correlated pleiotropy may have been partly biasing the BMI-HNC estimate. CAUSE, which is more robust to correlated pleiotropy than the IVW method, further supported this hypothesis.

Our results are consistent with previous MR studies that suggest adiposity does not influence HNC risk[27–29]. Gormley et al.[28] did not find a genetically predicted effect of adiposity on combined oral and oropharyngeal cancer when investigating either BMI (OR=0.89 per 1-SD, 95% CI 0.72–1.09, p=0.26), WHR (OR=0.98 per 1-SD, 95% CI 0.74–1.29, p=0.88) or waist circumference (OR=0.73 per 1-SD, 95% CI 0.52–1.02, p=0.07) as risk factors. Similarly, a large two-sample MR study by Vithayathil et al.[29] including 367,561 UK Biobank participants (of which 1,983 were HNC cases) found no link between BMI and HNC risk (OR=0.98 per 1-SD higher BMI, 95% CI 0.93–1.02, p=0.35). Larsson et al.[27] meta-analysed Vithayathil et al.’s[29] findings with results obtained using FinnGen data to increase the sample size even further (N=586,353, including 2,109 cases), but still did not find a genetically predicted effect of BMI on HNC risk (OR=0.96 per 1-SD higher BMI, 95% CI 0.77–1.19, p=0.69).

In our study, we found some evidence that the genetically predicted effect of BMI on HNC risk was influenced by smoking. This could be due to the bidirectional relationship between smoking and adiposity reported in previous MR studies[17–22] or due to their shared genetic architecture[60, 61]. A strength of our study was that it was the first to exploit MVMR to disentangle the effects of BMI and smoking behaviour on the risk of HNC and its subsites. An advantage of our approach compared to conducting univariable MR analyses stratified by smoking status is that the former does not induce collider bias and provides estimates of direct effects irrespective of horizontal pleiotropy or mediation. Yet, we acknowledge the smoking behaviour traits used in our MVMR analyses likely capture more than just smoking, since some of the SNPs used to instrument these traits have been associated with risk-taking phenotypes and socioeconomic factors[62–65]. This places limits in the inferences that can be made about smoking in the context of mediation.

An important strength of our study was that the HEADSpAcE consortium GWAS used had a large sample size which conferred more statistical power to detect effects of adiposity on HNC risk compared to previous MR analyses[27–29], increasing one’s confidence in the null nature of the estimates. Furthermore, the availability of data on more HNC subsites, including oropharyngeal cancers by HPV status, allowed us to investigate the relationship between adiposity and HNC risk in more detail than previous MR studies which limited their subsite analyses to oral cavity and overall oropharyngeal cancers[28, 66]. This is relevant because distinct HNC subsites are known to have different aetiologies[67], although we did not find evidence of heterogeneity across subsites in our study.

We acknowledge that a major limitation of MR studies, including ours, is that several untestable assumptions are required to make accurate causal inferences. It is unlikely that our findings were influenced by weak instrument bias (i.e., violating the relevance assumption) because we used strong genetic instruments to proxy our adiposity traits. However, the independence assumption of no genetic confounding and the exclusion restriction assumption of no horizontal pleiotropy could have been violated. Furthermore, we were unable to explore potential non-linear causal effects of adiposity on HNC risk in the present study.

While our study contributes valuable evidence on the role of adiposity in the development of HNC, we recognise there is a need for additional research on the subject. Our study was limited to individuals of European ancestry, so our findings should be replicated in other ancestry groups before being generalised to non-European populations. Moreover, further research is needed to understand the biology underlying the complex relationship between smoking and adiposity, especially since it may be difficult to intervene on one without influencing the other[17].

## Conclusions

In conclusion, this study indicates that adiposity does not play a role in HNC risk. Although we did not find strong evidence of a causal effect of adiposity on HNC, obesity is an established risk factor for multiple cancers and other chronic diseases[27, 68, 69]. Hence there is still value in aiming to reduce the levels of excess adiposity in the population.

## Supplementary Material

Supplement 1

Supplement 2

Supplement 3

## Figures and Tables

**Table 1.** Data sources and instruments for other adiposity-related anthropometric measures.

Study	Year	Data source	Trait	Unit	Download link or OpenGWAS ID
Ried et al.[56]	2016	GIANT	Body shape PC1 (overall adiposity)	SD	https://www.joelhirschhornlab.org/giant-consortium-results
			Body shape PC2 (tall and slim vs short and plump)	SD	
			Body shape PC3 (tall with small hip vs short with big hip)	SD	
			Body shape PC4 (high BMI and weight with small hip and waist vs low BMI and weight with big hip and waist)	SD	
Richardson et al.[57]	2020	UKB	Childhood body size	Change in body size category	"ieu-b-5107"
			Adulthood body size	Change in body size category	"ieu-b-5118"
Martin et al.[58]	2021	UKB	Metabolically favourable adiposity	SD	https://doi.org/10.2337/figshare.14555463.v1
			Metabolically unfavourable adiposity	SD	
MRC-IEU (Elsworth)	2018	UKB	Body fat percentage	SD	"ukb-b-8909"
Leyden et al.[59]	2022	GIANT + UKB	Brain tissue-specific BMI	SD	https://www.ncbi.nlm.nih.gov/pmc/articles/PMC8874216/bin/mmc2.xlsx
			Adipose tissue-specific BMI	SD	

Abbreviations: BMI, body mass index; GIANT, Genetic Investigation of Anthropometric Traits; N, number; PC, principal component; SD, standard deviation; SNP, single-nucleotide polymorphism; UKB, UK Biobank.

**Table 2.** F-statistics and variance explained for other adiposity-related anthropometric measures.

Trait	N SNPs before/after harmonisation	Total R^2^	Mean F-statistics (range)
Body shape PC1 (overall adiposity)	29/28	16%	54 (28–302)
Body shape PC2 (tall and slim vs short and plump)	84/81	3.4%	54 (30–211)
Body shape PC3 (tall with small hip vs short with big hip)	28/27	0.9%	41 (30–82)
Body shape PC4 (high BMI and weight with small hip and waist vs low BMI and weight with big hip and waist)	10/10	24.7%	42 (30–98)
Childhood body size	206/198	3.4%	78 (28–1102)
Adulthood body size	339/324	4.2%	59 (30–1109)
Metabolically favourable adiposity	34/31	0.4%	64 (25–400)
Metabolically unfavourable adiposity	29/27	0.8%	131 (25–400)
Body fat percentage	377/360	4.7%	59 (30–682)
Brain tissue-specific BMI	140/133	1.2%	61 (29–270)
Adipose tissue-specific BMI	86/81	0.7%	63 (30–270)

Abbreviations: BMI, body mass index; N, number; PC, principal component; SNP, single-nucleotide polymorphism.

## Data Availability

All the GWAS datasets used in our study are publicly available. The GWAS summary statistics for waist circumference are available via the IEU OpenGWAS platform (id: ukb-b-9405). The GWAS summary statistics for BMI and WHR by Pulit et al.[35] can be downloaded from https://zenodo.org/records/1251813. The data sources for the other adiposity-related measures have been specified in Table 1. The smoking behaviour traits GWAS data were downloaded from https://data.bris.ac.uk/data/dataset/10i96zb8gm0j81yz0q6ztei23d (for CSI) and https://doi.org/10.13020/przg-dp88 (for smoking initiation). The outcome datasets used in our analyses have been uploaded to the IEU OpenGWAS project platform for reproducibility. However, because the data was originally in build GRCh38, some multiallelic SNPs that could not be aligned with GRCh37 Human Genome reference sequence were dropped when lifting the data to build HG19/GRCh37 (which was required at the time of upload: April 2024). The following IEU OpenGWAS id’s were assigned to the European HEADSpAcE HNC GWAS datasets including/excluding UK Biobank: ieu-b-5129/ieu-b-5123 for overall HNC, ieu-b-5132/ieu-b-5126 for oral cavity cancer, ieu-b-5130/ieu-b-5124 for hypopharynx cancer, ieu-b-5134/ieu-b-5128 for HPV positive oropharynx cancer, ieu-b-5133/ieu-b-5127 for HPV negative oropharynx cancer, and ieu-b-5131/ieu-b-5125 for larynx cancer. The R code used to run the MR analyses is available at http://github.com/fernandam93/adiposity_HNC_MR.

## References

[1] Global Cancer Observatory: Cancer Today (version 1.1) [https://gco.iarc.who.int/today]

[2] Curado MP, Hashibe M: Recent changes in the epidemiology of head and neck cancer. Curr Opin Oncol 2009, 21(3):194–200. 19363341 10.1097/CCO.0b013e32832a68ca

[3] Hashibe M, Brennan P, Benhamou S, Castellsague X, Chen C, Curado MP, Dal Maso L, Daudt AW, Fabianova E, Fernandez L : Alcohol drinking in never users of tobacco, cigarette smoking in never drinkers, and the risk of head and neck cancer: pooled analysis in the International Head and Neck Cancer Epidemiology Consortium. J Natl Cancer Inst 2007, 99(10):777–789. 17505073 10.1093/jnci/djk179

[4] Lee YC, Boffetta P, Sturgis EM, Wei Q, Zhang ZF, Muscat J, Lazarus P, Matos E, Hayes RB, Winn DM : Involuntary smoking and head and neck cancer risk: pooled analysis in the International Head and Neck Cancer Epidemiology Consortium. Cancer Epidemiol Biomarkers Prev 2008, 17(8):1974–1981. 18708387 10.1158/1055-9965.EPI-08-0047 PMC2561190

[5] Purdue MP, Hashibe M, Berthiller J, La Vecchia C, Dal Maso L, Herrero R, Franceschi S, Castellsague X, Wei Q, Sturgis EM : Type of alcoholic beverage and risk of head and neck cancer--a pooled analysis within the INHANCE Consortium. Am J Epidemiol 2009, 169(2):132–142. 19064644 10.1093/aje/kwn306 PMC2727255

[6] Hashibe M, Brennan P, Chuang SC, Boccia S, Castellsague X, Chen C, Curado MP, Dal Maso L, Daudt AW, Fabianova E : Interaction between tobacco and alcohol use and the risk of head and neck cancer: pooled analysis in the International Head and Neck Cancer Epidemiology Consortium. Cancer Epidemiol Biomarkers Prev 2009, 18(2):541–550. 19190158 10.1158/1055-9965.EPI-08-0347 PMC3051410

[7] Marron M, Boffetta P, Zhang ZF, Zaridze D, Wunsch-Filho V, Winn DM, Wei Q, Talamini R, Szeszenia-Dabrowska N, Sturgis EM : Cessation of alcohol drinking, tobacco smoking and the reversal of head and neck cancer risk. Int J Epidemiol 2010, 39(1):182–196. 19805488 10.1093/ije/dyp291 PMC2817090

[8] Humans IWGotEoCRt: Tobacco smoke and involuntary smoking. IARC Monogr Eval Carcinog Risks Hum 2004, 83:1–1438. 15285078 PMC4781536

[9] Gormley M, Dudding T, Sanderson E, Martin RM, Thomas S, Tyrrell J, Ness AR, Brennan P, Munafo M, Pring M : A multivariable Mendelian randomization analysis investigating smoking and alcohol consumption in oral and oropharyngeal cancer. Nat Commun 2020, 11(1):6071. 33247085 10.1038/s41467-020-19822-6 PMC7695733

[10] Sabatini ME, Chiocca S: Human papillomavirus as a driver of head and neck cancers. Br J Cancer 2020, 122(3):306–314. 31708575 10.1038/s41416-019-0602-7 PMC7000688

[11] Gillison ML, Koch WM, Capone RB, Spafford M, Westra WH, Wu L, Zahurak ML, Daniel RW, Viglione M, Symer DE : Evidence for a causal association between human papillomavirus and a subset of head and neck cancers. J Natl Cancer Inst 2000, 92(9):709–720. 10793107 10.1093/jnci/92.9.709

[12] de Martel C, Georges D, Bray F, Ferlay J, Clifford GM: Global burden of cancer attributable to infections in 2018: a worldwide incidence analysis. Lancet Glob Health 2020, 8(2):e180–e190. 31862245 10.1016/S2214-109X(19)30488-7

[13] World Cancer Research Fund/American Institute for Cancer Research: Diet, nutrition, physical activity and cancers of the mouth, pharynx and larynx. In: Continuous Update Project Expert Report. 2018.

[14] Gaudet MM, Kitahara CM, Newton CC, Bernstein L, Reynolds P, Weiderpass E, Kreimer AR, Yang G, Adami HO, Alavanja MC : Anthropometry and head and neck cancer:a pooled analysis of cohort data. Int J Epidemiol 2015, 44(2):673–681. 26050257 10.1093/ije/dyv059 PMC4481608

[15] Jo YH, Talmage DA, Role LW: Nicotinic receptor-mediated effects on appetite and food intake. J Neurobiol 2002, 53(4):618–632. 12436425 10.1002/neu.10147 PMC2367209

[16] Watts EL, Moore SC, Gunter MJ, Chatterjee N: Adiposity and cancer: meta-analysis, mechanisms, and future perspectives. medRxiv 2024:10.1101/2024.1102.1116.24302944.

[17] Taylor AE, Richmond RC, Palviainen T, Loukola A, Wootton RE, Kaprio J, Relton CL, Davey Smith G, Munafo MR: The effect of body mass index on smoking behaviour and nicotine metabolism: a Mendelian randomization study. Hum Mol Genet 2019, 28(8):1322–1330. 30561638 10.1093/hmg/ddy434 PMC6452214

[18] Carreras-Torres R, Johansson M, Haycock PC, Relton CL, Davey Smith G, Brennan P, Martin RM: Role of obesity in smoking behaviour: Mendelian randomisation study in UK Biobank. BMJ 2018, 361:k1767. 29769355 10.1136/bmj.k1767 PMC5953237

[19] Asvold BO, Bjorngaard JH, Carslake D, Gabrielsen ME, Skorpen F, Smith GD, Romundstad PR: Causal associations of tobacco smoking with cardiovascular risk factors: a Mendelian randomization analysis of the HUNT Study in Norway. Int J Epidemiol 2014, 43(5):1458–1470. 24867305 10.1093/ije/dyu113

[20] Freathy RM, Kazeem GR, Morris RW, Johnson PC, Paternoster L, Ebrahim S, Hattersley AT, Hill A, Hingorani AD, Holst C : Genetic variation at CHRNA5-CHRNA3-CHRNB4 interacts with smoking status to influence body mass index. Int J Epidemiol 2011, 40(6):1617–1628. 21593077 10.1093/ije/dyr077 PMC3235017

[21] Taylor AE, Morris RW, Fluharty ME, Bjorngaard JH, Asvold BO, Gabrielsen ME, Campbell A, Marioni R, Kumari M, Hallfors J : Stratification by smoking status reveals an association of CHRNA5-A3-B4 genotype with body mass index in never smokers. PLoS Genet 2014, 10(12):e1004799. 25474695 10.1371/journal.pgen.1004799 PMC4256159

[22] Morris RW, Taylor AE, Fluharty ME, Bjorngaard JH, Asvold BO, Elvestad Gabrielsen M, Campbell A, Marioni R, Kumari M, Korhonen T : Heavier smoking may lead to a relative increase in waist circumference: evidence for a causal relationship from a Mendelian randomisation meta-analysis. The CARTA consortium. BMJ Open 2015, 5(8):e008808. 10.1136/bmjopen-2015-008808 PMC4538266 26264275

[23] Yang S, Lynch J, Schulenberg J, Diez Roux AV, Raghunathan T: Emergence of socioeconomic inequalities in smoking and overweight and obesity in early adulthood: the national longitudinal study of adolescent health. Am J Public Health 2008, 98(3):468–477. 18235067 10.2105/AJPH.2007.111609 PMC2253566

[24] Marmot M, Bell R: Social determinants and non-communicable diseases: time for integrated action. BMJ 2019, 364:l251. 30692093 10.1136/bmj.l251 PMC6348404

[25] Marmot M: The health gap: Doctors and the social determinants of health. Scand J Public Health 2017, 45(7):686–693. 29162019 10.1177/1403494817717448

[26] Filippidis FT, Agaku IT, Girvalaki C, Jimenez-Ruiz C, Ward B, Gratziou C, Vardavas CI, Tobacco Control Committee of the European Respiratory S: Relationship of secondhand smoke exposure with sociodemographic factors and smoke-free legislation in the European Union. Eur J Public Health 2016, 26(2):344–349. 26511601 10.1093/eurpub/ckv204

[27] Larsson SC, Burgess S: Causal role of high body mass index in multiple chronic diseases: a systematic review and meta-analysis of Mendelian randomization studies. BMC Med 2021, 19(1):320. 34906131 10.1186/s12916-021-02188-x PMC8672504

[28] Gormley M, Dudding T, Thomas SJ, Tyrrell J, Ness AR, Pring M, Legge D, Davey Smith G, Richmond RC, Vincent EE : Evaluating the effect of metabolic traits on oral and oropharyngeal cancer risk using Mendelian randomization. Elife 2023, 12. 10.7554/eLife.82674 PMC10147379 37042641

[29] Vithayathil M, Carter P, Kar S, Mason AM, Burgess S, Larsson SC: Body size and composition and risk of site-specific cancers in the UK Biobank and large international consortia: A mendelian randomisation study. PLoS Med 2021, 18(7):e1003706. 34324486 10.1371/journal.pmed.1003706 PMC8320991

[30] Lesseur C, Diergaarde B, Olshan AF, Wunsch-Filho V, Ness AR, Liu G, Lacko M, Eluf-Neto J, Franceschi S, Lagiou P : Genome-wide association analyses identify new susceptibility loci for oral cavity and pharyngeal cancer. Nat Genet 2016, 48(12):1544–1550. 27749845 10.1038/ng.3685 PMC5131845

[31] Davies NM, Holmes MV, Davey Smith G: Reading Mendelian randomisation studies: a guide, glossary, and checklist for clinicians. BMJ 2018, 362:k601. 30002074 10.1136/bmj.k601 PMC6041728

[32] Skrivankova VW, Richmond RC, Woolf BAR, Yarmolinsky J, Davies NM, Swanson SA, VanderWeele TJ, Higgins JPT, Timpson NJ, Dimou N : Strengthening the Reporting of Observational Studies in Epidemiology Using Mendelian Randomization: The STROBE-MR Statement. JAMA 2021, 326(16):1614–1621. 34698778 10.1001/jama.2021.18236

[33] Ebrahimi E, Sangphukieo A, Park H, Gaborieau V, Ferreiro-Iglesias A, Diergaarde B, Ahrens W, Alemany L, Arantes L, Betka J : Cross-ancestral GWAS identifies 29 novel variants across Head and Neck Cancer subsites. medRxiv 2024:10.1101/2024.1111.1118.24317473. PMC12491539 41038832

[34] World Health O, World Health O: ICD-10 : international statistical classification of diseases and related health problems, Tenth revision, Fifth edition. edn. Geneva: World Health Organization; 2016.

[35] Pulit SL, Stoneman C, Morris AP, Wood AR, Glastonbury CA, Tyrrell J, Yengo L, Ferreira T, Marouli E, Ji Y : Meta-analysis of genome-wide association studies for body fat distribution in 694 649 individuals of European ancestry. Hum Mol Genet 2019, 28(1):166–174. 30239722 10.1093/hmg/ddy327 PMC6298238

[36] Shungin D, Winkler TW, Croteau-Chonka DC, Ferreira T, Locke AE, Magi R, Strawbridge RJ, Pers TH, Fischer K, Justice AE : New genetic loci link adipose and insulin biology to body fat distribution. Nature 2015, 518(7538):187–196. 25673412 10.1038/nature14132 PMC4338562

[37] Loh PR, Tucker G, Bulik-Sullivan BK, Vilhjalmsson BJ, Finucane HK, Salem RM, Chasman DI, Ridker PM, Neale BM, Berger B : Efficient Bayesian mixed-model analysis increases association power in large cohorts. Nat Genet 2015, 47(3):284–290. 25642633 10.1038/ng.3190 PMC4342297

[38] Willer CJ, Li Y, Abecasis GR: METAL: fast and efficient meta-analysis of genomewide association scans. Bioinformatics 2010, 26(17):2190–2191. 20616382 10.1093/bioinformatics/btq340 PMC2922887

[39] Hemani G, Zheng J, Elsworth B, Wade KH, Haberland V, Baird D, Laurin C, Burgess S, Bowden J, Langdon R : The MR-Base platform supports systematic causal inference across the human phenome. Elife 2018, 7. 10.7554/eLife.34408 PMC5976434 29846171

[40] Palmer TM, Lawlor DA, Harbord RM, Sheehan NA, Tobias JH, Timpson NJ, Davey Smith G, Sterne JA: Using multiple genetic variants as instrumental variables for modifiable risk factors. Stat Methods Med Res 2012, 21(3):223–242. 21216802 10.1177/0962280210394459 PMC3917707

[41] Burgess S, Thompson SG: Bias in causal estimates from Mendelian randomization studies with weak instruments. Stat Med 2011, 30(11):1312–1323. 21432888 10.1002/sim.4197

[42] Burgess S, Butterworth A, Thompson SG: Mendelian randomization analysis with multiple genetic variants using summarized data. Genet Epidemiol 2013, 37(7):658–665. 24114802 10.1002/gepi.21758 PMC4377079

[43] Bowden J, Davey Smith G, Burgess S: Mendelian randomization with invalid instruments: effect estimation and bias detection through Egger regression. Int J Epidemiol 2015, 44(2):512–525. 26050253 10.1093/ije/dyv080 PMC4469799

[44] Bowden J, Davey Smith G, Haycock PC, Burgess S: Consistent Estimation in Mendelian Randomization with Some Invalid Instruments Using a Weighted Median Estimator. Genet Epidemiol 2016, 40(4):304–314. 27061298 10.1002/gepi.21965 PMC4849733

[45] Hartwig FP, Davey Smith G, Bowden J: Robust inference in summary data Mendelian randomization via the zero modal pleiotropy assumption. Int J Epidemiol 2017, 46(6):1985–1998. 29040600 10.1093/ije/dyx102 PMC5837715

[46] Bowden J, Del Greco MF, Minelli C, Zhao Q, Lawlor DA, Sheehan NA, Thompson J, Davey Smith G: Improving the accuracy of two-sample summary-data Mendelian randomization: moving beyond the NOME assumption. Int J Epidemiol 2019, 48(3):728–742. 30561657 10.1093/ije/dyy258 PMC6659376

[47] Verbanck M, Chen CY, Neale B, Do R: Detection of widespread horizontal pleiotropy in causal relationships inferred from Mendelian randomization between complex traits and diseases. Nat Genet 2018, 50(5):693–698. 29686387 10.1038/s41588-018-0099-7 PMC6083837

[48] Sanderson E, Davey Smith G, Windmeijer F, Bowden J: An examination of multivariable Mendelian randomization in the single-sample and two-sample summary data settings. Int J Epidemiol 2019, 48(3):713–727. 30535378 10.1093/ije/dyy262 PMC6734942

[49] Saunders GRB, Wang X, Chen F, Jang S-K, Liu M, Wang C, Gao S, Jiang Y, Khunsriraksakul C, Otto JM : Genetic diversity fuels gene discovery for tobacco and alcohol use. Nature 2022, 612(7941):720–724. 36477530 10.1038/s41586-022-05477-4 PMC9771818

[50] Wootton RE, Richmond RC, Stuijfzand BG, Lawn RB, Sallis HM, Taylor GMJ, Hemani G, Jones HJ, Zammit S, Davey Smith G : Evidence for causal effects of lifetime smoking on risk for depression and schizophrenia: a Mendelian randomisation study. Psychol Med 2020, 50(14):2435–2443. 31689377 10.1017/S0033291719002678 PMC7610182

[51] Darrous L, Mounier N, Kutalik Z: Simultaneous estimation of bi-directional causal effects and heritable confounding from GWAS summary statistics. Nat Commun 2021, 12(1):7274. 34907193 10.1038/s41467-021-26970-w PMC8671515

[52] Morrison J, Knoblauch N, Marcus JH, Stephens M, He X: Mendelian randomization accounting for correlated and uncorrelated pleiotropic effects using genome-wide summary statistics. Nat Genet 2020, 52(7):740–747. 32451458 10.1038/s41588-020-0631-4 PMC7343608

[53] Hemani G, Tilling K, Davey Smith G: Orienting the causal relationship between imprecisely measured traits using GWAS summary data. PLoS Genet 2017, 13(11):e1007081. 29149188 10.1371/journal.pgen.1007081 PMC5711033

[54] Sanderson E, Rosoff D, Palmer T, Tilling K, Smith GD, Hemani G: Bias from heritable confounding in Mendelian randomization studies. medRxiv 2024:2024.2009.2005.24312293.

[55] Foley CN, Mason AM, Kirk PDW, Burgess S: MR-Clust: clustering of genetic variants in Mendelian randomization with similar causal estimates. Bioinformatics 2021, 37(4):531–541. 32915962 10.1093/bioinformatics/btaa778 PMC8088327

[56] Ried JS, Jeff MJ, Chu AY, Bragg-Gresham JL, van Dongen J, Huffman JE, Ahluwalia TS, Cadby G, Eklund N, Eriksson J : A principal component meta-analysis on multiple anthropometric traits identifies novel loci for body shape. Nat Commun 2016, 7:13357. 27876822 10.1038/ncomms13357 PMC5114527

[57] Richardson TG, Sanderson E, Elsworth B, Tilling K, Davey Smith G: Use of genetic variation to separate the effects of early and later life adiposity on disease risk: mendelian randomisation study. BMJ 2020, 369:m1203. 32376654 10.1136/bmj.m1203 PMC7201936

[58] Martin S, Cule M, Basty N, Tyrrell J, Beaumont RN, Wood AR, Frayling TM, Sorokin E, Whitcher B, Liu Y : Genetic Evidence for Different Adiposity Phenotypes and Their Opposing Influences on Ectopic Fat and Risk of Cardiometabolic Disease. Diabetes 2021, 70(8):1843–1856. 33980691 10.2337/db21-0129

[59] Leyden GM, Shapland CY, Davey Smith G, Sanderson E, Greenwood MP, Murphy D, Richardson TG: Harnessing tissue-specific genetic variation to dissect putative causal pathways between body mass index and cardiometabolic phenotypes. Am J Hum Genet 2022, 109(2):240–252. 35090585 10.1016/j.ajhg.2021.12.013 PMC8874216

[60] Thorgeirsson TE, Gudbjartsson DF, Sulem P, Besenbacher S, Styrkarsdottir U, Thorleifsson G, Walters GB, Consortium TAG, Oxford GSKC, consortium E : A common biological basis of obesity and nicotine addiction. Transl Psychiatry 2013, 3(10):e308. 24084939 10.1038/tp.2013.81 PMC3818010

[61] Wills AG, Hopfer C: Phenotypic and genetic relationship between BMI and cigarette smoking in a sample of UK adults. Addict Behav 2019, 89:98–103. 30286397 10.1016/j.addbeh.2018.09.025 PMC6240387

[62] Gage SH, Sallis HM, Lassi G, Wootton RE, Mokrysz C, Davey Smith G, Munafo MR: Does smoking cause lower educational attainment and general cognitive ability? Triangulation of causal evidence using multiple study designs. Psychol Med 2022, 52(8):1578–1586. 33023701 10.1017/S0033291720003402 PMC9226381

[63] Schellhas L, Haan E, Easey KE, Wootton RE, Sallis HM, Sharp GC, Munafo MR, Zuccolo L: Maternal and child genetic liability for smoking and caffeine consumption and child mental health: an intergenerational genetic risk score analysis in the ALSPAC cohort. Addiction 2021, 116(11):3153–3166. 33891774 10.1111/add.15521 PMC9376939

[64] Khouja JN, Wootton RE, Taylor AE, Davey Smith G, Munafo MR: Association of genetic liability to smoking initiation with e-cigarette use in young adults: A cohort study. PLoS Med 2021, 18(3):e1003555. 33735204 10.1371/journal.pmed.1003555 PMC7971530

[65] Reed ZE, Wootton RE, Khouja JN, Richardson TG, Sanderson E, Davey Smith G, Munafo MR: Exploring pleiotropy in Mendelian randomisation analyses: What are genetic variants associated with ‘cigarette smoking initiation’ really capturing? Genet Epidemiol 2024. 10.1002/gepi.22583 PMC7616876 39099143

[66] Gui L, He X, Tang L, Yao J, Pi J: Obesity and head and neck cancer risk: a mendelian randomization study. BMC Med Genomics 2023, 16(1):200. 37620971 10.1186/s12920-023-01634-4 PMC10463997

[67] Thomas SJ, Penfold CM, Waylen A, Ness AR: The changing aetiology of head and neck squamous cell cancer: A tale of three cancers? Clin Otolaryngol 2018, 43(4):999–1003. 29770611 10.1111/coa.13144

[68] Lauby-Secretan B, Scoccianti C, Loomis D, Grosse Y, Bianchini F, Straif K, International Agency for Research on Cancer Handbook Working G: Body Fatness and Cancer--Viewpoint of the IARC Working Group. N Engl J Med 2016, 375(8):794–798. 27557308 10.1056/NEJMsr1606602 PMC6754861

[69] Mariosa D, Carreras-Torres R, Martin RM, Johansson M, Brennan P: Commentary: What can Mendelian randomization tell us about causes of cancer? Int J Epidemiol 2019, 48(3):816–821. 31503317 10.1093/ije/dyz151 PMC6659369

